# Using graph learning to understand adverse pregnancy outcomes and stress pathways

**DOI:** 10.1371/journal.pone.0223319

**Published:** 2019-09-30

**Authors:** Octavio Mesner, Alex Davis, Elizabeth Casman, Hyagriv Simhan, Cosma Shalizi, Lauren Keenan-Devlin, Ann Borders, Tamar Krishnamurti

**Affiliations:** 1 Department of Engineering and Public Policy, Carnegie Mellon University, Pittsburgh, PA, United States of America; 2 Department of Statistics and Data Science, Carnegie Mellon University, Pittsburgh, PA, United States of America; 3 Magee-Women’s Research Institute, Pittsburgh, PA, United States of America; 4 Northshore University Health System, Evanston, Illinois, United States of America; 5 Department of General Internal Medicine, University of Pittsburgh School of Medicine, Pittsburgh, PA, United States of America; Holbæk Hospital, DENMARK

## Abstract

To identify pathways between stress indicators and adverse pregnancy outcomes, we applied a nonparametric graph-learning algorithm, PC-KCI, to data from an observational prospective cohort study. The Measurement of Maternal Stress study (MOMS) followed 744 women with a singleton intrauterine pregnancy recruited between June 2013 and May 2015. Infant adverse pregnancy outcomes were prematurity (<37 weeks' gestation), infant days spent in hospital after birth, and being small for gestational age (percentile gestational weight at birth). Maternal adverse pregnancy outcomes were pre-eclampsia, gestational diabetes, and gestational hypertension. PC-KCI replicated well-established pathways, such as the relationship between gestational weeks and preterm premature rupture of membranes. PC-KCI also identified previously unobserved pathways to adverse pregnancy outcomes, including 1) a link between hair cortisol levels (at 12–21 weeks of pregnancy) and pre-eclampsia; 2) two pathways to preterm birth depending on race, with one linking Hispanic race, pre-gestational diabetes and gestational weeks, and a second pathway linking black race, hair cortisol, preeclampsia, and gestational weeks; and 3) a relationship between maternal childhood trauma, perceived social stress in adulthood, and low weight for gestational age. Our approach confirmed previous findings and identified previously unobserved pathways to adverse pregnancy outcomes. It presents a method for a global assessment of a clinical problem for further study of possible causal pathways.

## Introduction

The biological and psychosocial pathways leading to adverse pregnancy outcomes, such as preterm birth, are complex and only partially understood. In this work, we show how to use a graph learning algorithm, creating a diagram of variables connected by statistical associations, to model those pathways simultaneously, providing an interpretable high-level view of potential causal mechanisms. The approach can be applied to many medical domains to help the design of future clinical studies, by determining promising variables for intervention, variables that should be measured to avoid confounding, and variables that are not predictive when other more causally proximal variables are measured.

Adverse pregnancy outcomes are a well-studied topic, where researchers have examined a range of potential causes, from psychosocial factors such as stress, childhood neglect, and depression, to biological indicators such as inflammation, hypertension, and diabetes. A substantial body of work has focused on the relationship between stress and adverse pregnancy outcomes [1–5], although the evidence from observational studies on the relationship is mixed. For example, a 2003 study found that anxiety, negative life events, and perceived racial discrimination were all associated with increased risk of preterm birth [6]. Likewise, two studies from 1996 and 2018 found that stress was associated with both preterm birth and low birth weight [7–8]. However, another found that only anxiety, out of many stressors and psychological distress measures, was associated with preterm birth [9]. Similarly, a 2017 study found no association between depression and preterm birth [10], and a 2008 study was not able to predict preterm birth from anxiety and perceived stress measured at 18–20 and 30–32 weeks of gestation [11].

Although adverse pregnancy outcomes likely result from many factors that work in concert, research typically focuses on single pathways or a few factors. While incremental knowledge is critical for building a foundational body of literature, such an approach risks missing complex interrelationships between many variables, and may fail to control for relevant confounders as research evolves. For example, most studies use variants of the generalized linear model to estimate the relationship between risk factors and a single adverse pregnancy outcome, but this approach cannot capture pathways that depend on a cascade of risk factors resulting in a clinical event. To allow a more comprehensive view to emerge, we apply a *nonparametric graph-learning algorithm*, which we call PC-KCI (after the PC [12] and Kernel Conditional Independence [13] algorithms), to “learn” or estimate a probabilistic graphical model based on data from the Measures of Maternal Stress (MOMS) study, an observational prospective cohort study. The MOMS data consist of multiple adverse pregnancy outcomes—both maternal (e.g., preeclampsia) and infant (e.g., less than 37 weeks of gestation)—and cover a variety of stress risk factors, ranging from pregnancy-related anxiety to stress biomarkers. The resultant graph represents all pairwise associations not mediated through other variables in the dataset, allowing researchers to examine 1) potential pathways from indicators to adverse pregnancy outcomes that may be useful for prediction or intervention, and 2) those variables that play an indirect role (or no role) in clinical pathways. We illustrate how the approach provides a model for a global assessment of a clinical problem in a simple visual representation of variable relationships for further study of possible causal pathways.

## Materials and methods

### Data

In the MOMS Study, 744 women were recruited between June 2013 and May 2015 from four sites, Northwestern University, University of Texas Health Science Center at San Antonio, University of Pittsburgh, and Schuylkill County, Pennsylvania, a rural site led by Children’s Hospital of Philadelphia. All women were at least 18 years of age with a singleton intrauterine pregnancy, less than 21 weeks pregnant at enrollment, English-speaking, and with no known fetal congenital anomalies. Enrolled women were examined twice, once between 12 and 21 weeks of gestation (visit A), and again between 32 and 36 weeks of gestation (visit B). Due to a higher proportion of missing data, including some key outcomes, we did not use variables collected at visit B. In all, there were 744 women at visit A and 639 at visit B; ultimately, 686 post-delivery medical records, such as pregnancy outcomes, were available. Additional details about the original data collection can be found in prior publications [14]. Ethics approval for the original data collection was provided by the Institutional Review Board of Northwestern University in Evanston IL, project number STU00039484. Participants gave informed consent before taking part. The current study is a secondary analysis of deidentified data.

With the help of stress and pregnancy experts, we removed variables that were redundant (e.g., a linear transformation of two biomarkers) or known to be irrelevant (e.g., participant study ID) from the analysis, leaving the following variables: *stress biomarkers* (2 variables), *psychosocial factors* (8 variables), *maternal medical history* (8 variables), *demographics* (8 variables), *inflammatory biomarkers* (7 variables), *abuse and disadvantage* (3 variables), and *adverse outcomes* (8 variables). The adverse outcomes of interest included infant outcomes (i.e., number of days in the NICU, gestational weeks, percentile weight for gestational age) and maternal outcomes (i.e., gestational diabetes, gestational hypertension, preeclampsia, and C-section). Descriptions of each included variable are in Table 1 and patient characteristics are summarized in Table 2.

**Table 1:** Variables from the Measures of Maternal Stress (MOMS) study included in the graphical model.

Variable Name	Variable Description	Mean (SD) or %
Age	Age (calculated from birthday and enrollment form date)	29.2 (5.7) years
Black	Black maternal race	17.2%
BMI	Body Mass Index: Weight*4.88/(Height^2)	27 (7.4)
C-section	Cesarean section	13.2%
Childhood abuse	Total score on the Questions about Your Childhood measure (Wadhwa, Buss, Entringer)	13.2 (2.8)
Childhood Disadvantage	Maternal Childhood Disadvantage variable, including whether the family owned a home, obtained medical treatment when necessary, received public assistance, purchased new clothes on special occasions, and owned a car, television, and washer and dryer (mean of 8 items)	1.0 (1.3)
Childhood Trauma	Total summed score for Emotional Abuse subscale, Emotional Neglect subscale, Physical Abuse subscale, Physical Neglect subscale, and Sexual Abuse subscale from the Childhood Trauma Questionnaire (Bernstein 1994)	Emotional abuse 8.24 (4.4) Emotional neglect 8.5 (4.1) Physical abuse 7.0 (3.6) Physical neglect 6.3 (2.5) Sexual abuse 7.0 (4.7)
CRH	Average Corticotropin Releasing Hormone (pg/mL)	23.1 (22.7)
C-Reactive Protein	C-reactive protein (mg/L)	8.0 (7.7)
Days NICU	Number of days in neonatal ICU	2.0 (8.7)
Depression Anxiety Rx Meds	Depression or anxiety medication taken during the 3 months prior to pregnancy	5.7%
Depression	Total score on the Center for Epidemiological Studies–Depression Scale (Radloff, 1977)	13.7 (10.6)
Discrimination	Total score on the Williams discrimination scale (Williams, 1997)	13.3 (5.7)
Domestic Abuse	Total score on the Abuse Assessment Screen (McFarlane, 1992)	13.2 (2.8)
EBV IgG	Epstein-Barr virus antibody	299.5 (235.5)
Education	Maternal self-reported education level	High school only 26.7% Some college 34.2% Bachelor’s and more 39%
Gestational Hypertension	Gestational hypertension	11.2%
Gestational weeks	Gestational age at delivery (in weeks) *Note*: <37 weeks’ gestation is used as a cut-off for prematurity	38.9 (2.1)
Gestational Diabetes	Gestational diabetes	8.2%
Hair cortisol	Hair Cortisol measure (pg/ml)	37.0 (234.4)
Hispanic and other	Hispanic or other maternal race	24.9%
IFN gamma	Interferon Gamma (pg/mL)	6.2 (38.2)
IL6	Interleukin 6 (pg/mL)	0.7 (1.6)
IL8	Interleukin 8 (pg/mL)	4.2 (58.6)
IL10	Interleukin 10 (pg/mL)	0.44 (1.2)
IL13	Interleukin 13 (pg/mL)	3.9 (3.9)
Income	Total income categorized into 4 groups	$0 - $15,000 108 16.2% $15,000-$50,000 33.1% $50,000 - $100,000 28.9% $100,000+ 146 21.9%
Insurance type	Maternal self-reported health insurance or healthcare coverage in past 12 months	Private 58.9% Other 41.1%
Married	Maternal self-reported marital status	81.1%
Maternal Weight Gain	Weight gain during pregnancy divided by gestational age (in weeks) at delivery.	0.95 (0.52)
Number of Previous Births	How many times the patient has given live birth	0.97 (1.2)
Number of Pregnancies	Including current pregnancy, how many times the patient has been pregnant, including miscarriage, stillbirth, etc.	2.5 (1.2)
Perceived Social Stress	Total score on the Cohen Perceived Social Stress Questionnaire (Cohen 1983)	15.7 (7.0)
Percentile Gestational Birthweight	Percentile rank pertaining to the infant's weight at birth, specific to that infant's gestational age at birth, mother's parity, and infant's sex *Note*: birthweights below the 10th percentile are used as the cutoff for Small for gestational age	0.50 (0.28)
PPROM	Premature rupture of membranes	20 (2.9)
Preeclampsia	Preeclampsia / eclampsia	5.2%
Pregestational diabetes	Pre-gestational diabetes taken from delivery records	56 (8.2)
Prenatal Distress	Total score on the Prenatal Distress questionnaire (Yani & Lobel, 1999)	13.5 (7.7)
Pre-pregnancy Rx meds	Non-depression or anxiety prescription medications taken in the 3 months prior to pregnancy, including medication for Sleep, Indigestion/ Heartburn, Asthma, Severe Headaches/ Migraines, Blood Sugar, Blood Pressure, Fertility (clomid, letrasol), or Antibiotics	42%
Prior Birth Preterm	Prior preterm birth	Never given birth 43.6% Only full-term deliveries 49.5% At least one preterm delivery 6.9%
Self Esteem	Total score on the Self-Esteem, Mastery, and Optimism subscales (Rosenberg, 1965)	74.3 (9.6)
Sleep quality	Total score on the Sleep Quality Index (Buysse, 1989)	5.3 (2.6)
Smoke	Pre-pregnancy maternal smoking status	10.2%
Social Problems	Total score on the Social Problems Questionnaire (Corney, 1985)	12.5 (7.0)
Social Support	Total score on the Social support questionnaire (Sherbourne and Stewart, 1991) at visit A	76.9 (12.9)
TNF alpha	Tumor necrosis factor alpha (pg/mL)	1.1 (1.8)
White	White maternal race	58.0%

**Table 2:** Attributes of women in the Measures of Maternal Stress (MOMS) study.

Variable	Frequency (%) or Mean (Range)
**Participants**	744^a^
**Hospital**	
Children’s Hospital of Philadelphia	175 (24%)
Northwestern University	191 (26%)
University of Pittsburgh	200 (27%)
University of Texas Health Science Center at San Antonio	178 (24%)
**Age, mean (IQR)**	29 (25, 33) years
**Race**	
Black	127 (17%)
Hispanic / Latino	145 (20%)
Non-Hispanic White	145 (58%)
Other	39 (5%)
**Income**	
<15k	108 (16%)
15–50k	221 (33%)
50–100k	193 (29%)
>100k	146 (22%)
**Education**	
Less than high school	198 (27%)
High school / GED	254 (34%)
Some college	289 (34%)
2-year college degree (Associate’s)	36 (5%)
Refused to answer	1 (<1%)
**Maternal adverse events**	
Preeclampsia	36 (5%)
Gestational Hypertension	77 (11%)
Pre-gestational Diabetes	24 (3%)
Gestational Diabetes	56 (8%)
**Infant adverse events**	
Infant in Hospital	2 (2,3) days
Neonatal Intubation	21 (3%)
Preterm birth	57 (8%)

^a^ No subjects were excluded from the analysis, although some subjects were missing information for specific variables.

### Patient involvement

This was a secondary analysis and no patients were directly involved in the research presented here. In the original data collection, patients were not directly involved in the process of study design, recruitment, or conduct. Results were shared in presentations at the participating institutions, and all findings and data were made public due to their federal funding status. All results presented are aggregated across patients and patients are not individually-identified.

### PC-KCI algorithm

PC-KCI is based on a graph-learning algorithm, called the *PC algorithm* [12], which performs a systematic series of conditional independence tests to construct a graph that represents the statistical dependence relationships (relationships that cannot be explained by other measured variables) between variables in a dataset. We use a novel implementation of *kernel conditional independence* (KCI) [13] testing with the *PC algorithm*, which we call PC-KCI, to establish connections (called an *edge* in the graphical models literature) in the graph [12]. KCI enables the detection of any statistical dependency, including nonlinear relationships, between variables in the MOMS dataset.

**Fig 1:**
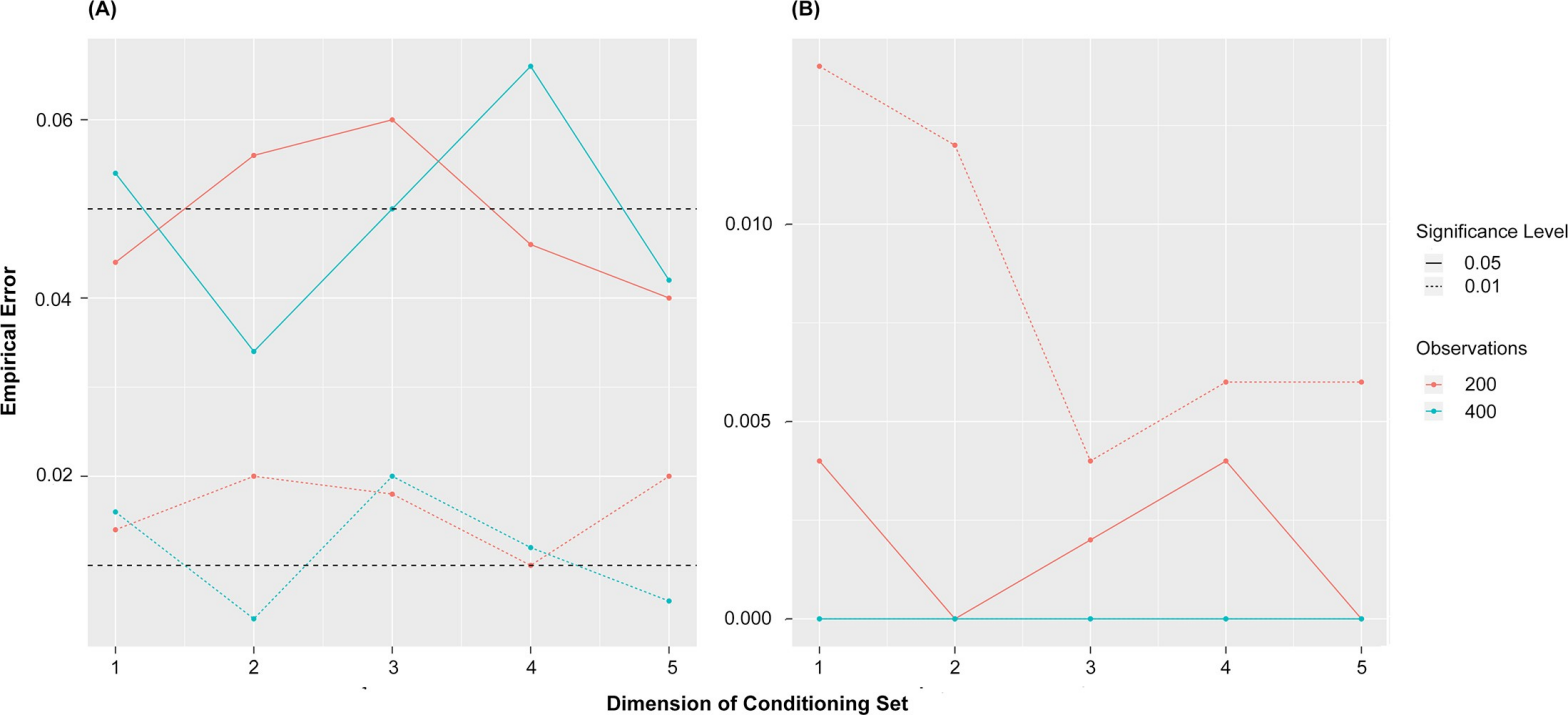
Type I error rate (A) & Type II error rate (B) for the KCI test varying sample size (200 and 400) and number of variables (2–5).

KCI uses a mathematical framework developed in functional analysis called the reproducing kernel Hilbert space (RKHS). Loosely, RKHS can analyze correlations of random variables under smooth transformations, allowing for the detection of non-linear statistical dependence. KCI’s test statistic uses a metric defined on the RKHS that is zero if and only if all transformations of the random variables are uncorrelated and corresponds to independence with a sufficiently rich set of transformations. Zhang *et al*. [13] showed how to estimate this metric from data and that the test statistic under the null hypothesis (conditional independence) can be approximated using the gamma distribution. As part of this work, we translated this theory into code in the R programming language (an R package will be published in 2019). To verify that our KCI code performed properly, we executed it on 500 simulated datasets of each size and dimension indicated in Fig 1. To test for type I error (Fig 1A), we generated conditionally independent data and plotted the proportion of times the test was (incorrectly) significant at the indicated significance level. To test type II error (Fig 1B), we generated non-linear dependent data and plotted the error rate of the test again. In general, this number should be small, but there are no predefined significance value as with type I error. The plot shows strong performance on both metrics.

Using the KCI test, the algorithm begins by connecting all variables (represented as nodes or vertices in the graph), then removes edges between variables if they are *marginally independent*—i.e., not associated according to a test of statistical independence. In the next step, the algorithm sequentially controls for other sets of variables that could explain the dependence between the two variables. If two variables that were initially connected after the first step (testing for marginal independence) become conditionally independent after the second step (controlling for one or more other variables), the edge between the first pair of variables is removed. This can occur if a causal pathway is mediated through the conditioning variables, or if the conditioning variables influence the original two variables but the original two variables do not influence each other (they share a common cause). After iterating through all pairs of variables with reasonable combinations of control variables, any edges that remain between two variables indicate statistical dependence not explained by other variables, and can be visualized in graphical form, where an edge between two variables in the dataset (called *neighbors*) typically indicates either a direct causal relationship or a statistical association induced by an unmeasured variable (*confounding*). This undirected graph-building process creates the skeleton graph that underlies most causal discovery algorithms, e.g. PC and FCI [12]. These algorithms all assume faithfulness when constructing the undirected graph, which means that any causal relationships imply marginal statistical dependence. A violation of faithfulness would be if two causes of a third variable exactly cancel each other out (statistically). Going from an undirected (association) to direct (causal) graph requires an additional battery of logical tests to orient the edges (indicating the direction of causality). We choose to present the graph without directed edges because of potential confounding variables not measured in the MOMS dataset, making errors in the direction of causality likely. The undirected graph still contains potentially causal pathways but does not indicate the direction of causality.

**Fig 2:**
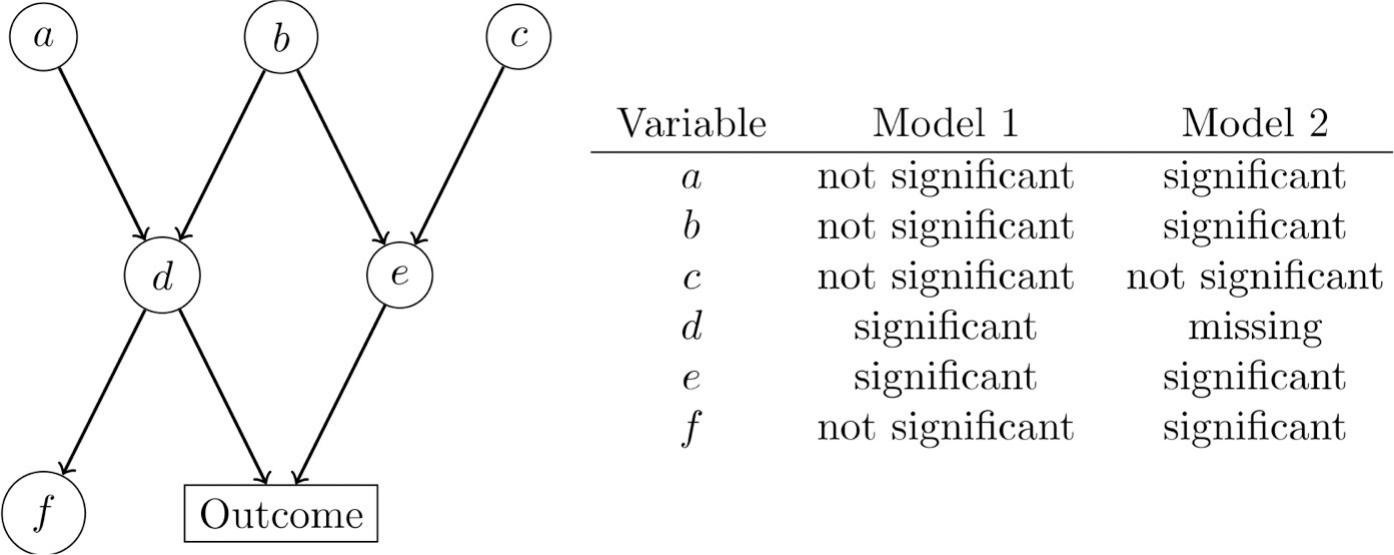
Relationship between graph structure and regression results. *Note*: The left-hand side shows the true underlying causal structure of a fictitious dataset with enough observations to detect significance. For example, a and b jointly influence d; d and e jointly influence the outcome. Further, there are no unmeasured variables affecting two or more variables in the data collected, variables a through f. The right-hand side shows the expected regression results given the underlying causal structure. Model 1 regresses the outcome on all variables, a through f. Notice that while a, b, and c are, in fact, indirect causes of the outcome through d and e, Model 1 renders these associations not significant because it is controlling for the mediating variables, d and e. Model 2 regresses the outcome on all variables except d, which is left out of the model. Without d, the model finds new associations with a, b, and f because that pathway is no longer blocked by d.

Fig 2 shows that the availability and use of differing control variables may, at least partly, explain differences between publications finding significance, even while using similar regression models. PC-KCI provides the high-level structure needed for understanding those differences, as well as potential causal pathways, whereas regression estimates specific functional relationships between a single outcome and its covariates. Their uses are complementary. For example, a graph constructed using PC-KCI can be used to understand mediation relations between two variables A and B connected in the graph after controlling for potential confounders C that are connected to both A and B. There are two important caveats with this approach: 1) this can only be done if the researcher is sure that both A and B do not cause C, and 2) statistics (p-values, confidence intervals) based on regression models that use the graph structure will not necessarily have their nominal levels of significance. For the latter reason we examine only the structure of the dependency relationships in the MOMs data, avoiding specific parametric models. An alternative approach would split the data into two parts, one for learning the structure with PC-KCI, the other for estimating parametric models given the structure. Another would be to use some sort of adjustment on the p-values and confidence intervals based on the number of tests used in the PC-algorithm, or possibly an approach similar to post-selection adjustment (although such an approach does not yet exist for the PC-algorithm) [15]. PC-KCI also has other uses, such as providing a general test of conditional association between two variables even when the specific functional relationship is not of interest, can suggest potential causal pathways, and can broadly cluster variables into groups to check whether they are highly (or loosely) associated.

## Results

**Fig 3:**
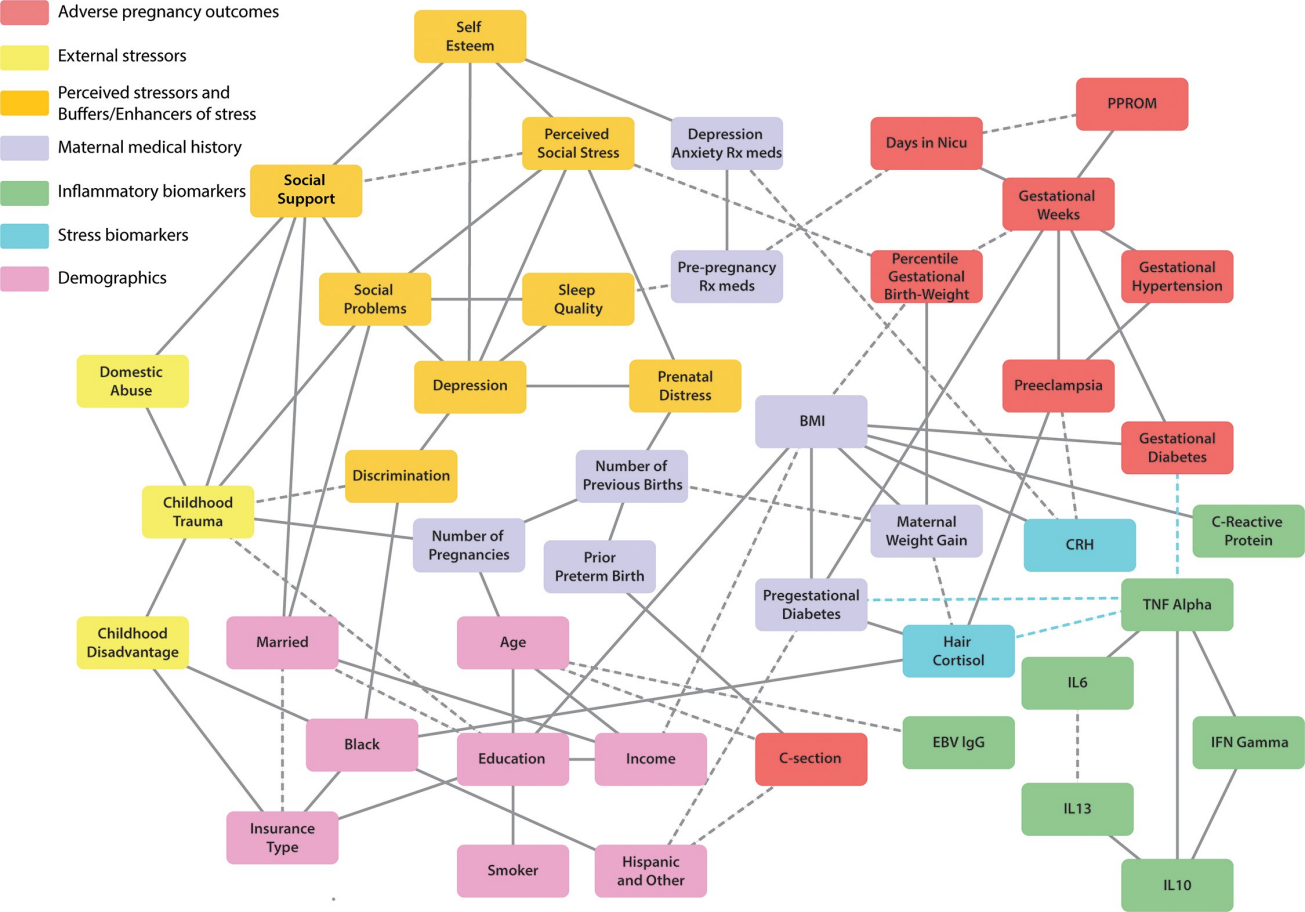
Graphical output from PC-KCI algorithm, identifying potential pathways from stress variables to adverse pregnancy outcomes for 4 US birth cohorts, 2013–2015. *Note*: Solid lines indicate p < .01 associations, while dashed lines indicate p < .05. Blue dots indicate an example pathway missed by PC-KCI (false negative results).

We ran PC-KCI twice, first only allowing edges for associations testing at *P* < 0.01 (Fig 3, solid lines); second, allowing edges for *P* < 0.05 (Fig 3, dashed lines, though solid if an edge was also in the P < 0.01 graph).

Links to the algorithms can be found in the supplemental material, **S1 Text** and **S2 Text.**

### Potential causal pathways

In analyzing the results of the algorithm, two types of relationships (or pathways) emerge—those that confirm prior research, and those that extend or add to prior knowledge. First, we examine relationships in the graph that confirm established prior research on adverse pregnancy outcomes. These variables are shaded red in Fig 2. The most obvious relationships are among the neighbors of infant adverse pregnancy outcomes, where gestational weeks is closely related to preterm premature rupture of membranes (PPROM), length of stay in the neonatal intensive care unit (NICU), and percentile gestational weight at birth. Similar patterns emerge between gestational weeks at birth and most maternal adverse outcomes (maternal gestational diabetes, preeclampsia, and gestational hypertension).

The positive relationship between BMI, C-Reactive Protein (CRP) and earlier gestational age at delivery confirms what has been established in the pregnancy literature [16–18]. Similarly, race, a documented risk factor for preterm birth [19–20], was connected to pre-gestational diabetes, which itself was connected to gestational weeks. In our findings, Hispanic vs White ethnicity was connected to pre-gestational diabetes which we know from the literature to be a population trend. Each of these relationships confirms what we know from many published papers and common knowledge of the clinical landscape.

The relationship between race and adverse pregnancy outcomes is nuanced, where pathways differ by race. African-American participants were more likely to have elevated hair cortisol levels, which, in turn, was associated with pre-eclampsia onset and shorter gestational weeks at birth, whereas Hispanic participants were more likely to exhibit pre-gestational diabetes leading to shorter gestational weeks. Also, prior preterm birth, maternal age, and Hispanic ethnicity were connected to whether a patient had a C-section, with Hispanic women in the sample almost twice as likely to have a C-section (20%) compared to non-Hispanic women (12%). The graph indicates that this relationship was not mediated by BMI (a commonly cited cause of C-sections), suggesting other possible explanations, such as patient preference for the procedure or variation between hospitals (e.g., San Antonio vs. Pittsburgh) in C-section rates.

The PC-KCI algorithm further found that higher scores on the Childhood Trauma Questionnaire were associated with lower percentile gestational weight at birth (i.e. infants who are small for gestational age) and, furthermore, that this was related to social problems and perceived social stress in adulthood.

Prior literature has shown a relationship between economically disadvantaged childhood and shorter gestational weeks, when controlling for current income [14]. Here we find that this outcome was related to current economic disadvantage, as indicated by insurance type (or lack thereof), suggesting that factors related to economic status that are distinct from income, may play a role in the preterm birth pathway. We also used PC-KCI to identify variables that were not *directly* statistically associated with each other, given the other variables used to create the graph, to inform future analyses for predicting adverse pregnancy outcomes. For example, many of the inflammatory biomarkers included were not found to directly connect to adverse pregnancy outcomes in this analysis. Existing literature on inflammatory biomarkers and adverse pregnancy outcomes suggests that these pathways are complex, and as in the case of CRP, are complicated by other factors such as adiposity. These findings indicate the importance of accounting for these indirect pathways between predictor and outcomes variables. Given the compelling evidence that inflammatory markers play a role in pre-term births, including IL-6 and TNF-alpha [21], this work adds to the literature by suggesting that these markers may operate through onset of medical conditions in pregnancy, such as gestational diabetes.

It is also important to note that some variables in the graph that are not connected by a pathway may still be statistically associated (a false negative result). Such an omitted edge is indicated by a dotted blue line in Fig 2. Forthcoming work has found that participants who fall in the top quartile of TNF-alpha serum concentration are more likely to experience preterm labor. PC-KCI did not detect this relationship, though TNF-alpha is marginally related to preterm birth. This is explained by the fact that when conditioning (controlling), for hair cortisol, gestational diabetes, and pre-gestational diabetes, the relationship between TNF-alpha and gestational weeks is no longer significant. PC-KCI is a conservative algorithm, prone to omitting edges rather than adding them, but in this case, the omission was due to the confounding influences of other variables in the putative pathway.

## Discussion

We found several important clinical results that suggest promising areas for prediction of adverse pregnancy outcomes. In particular, certain stress-related biopsychosocial variables were related to preeclampsia, including change in corticotropin releasing hormone and hair cortisol levels measured at 12–21 weeks of pregnancy. Detecting preeclampsia risk early in pregnancy may allow for early intervention using low-dose aspirin prophylaxis [22–24]. Additionally, some notable pathways related to race/ethnicity, such as Hispanic ethnicity being linked to greater levels of pre-gestational diabetes, which in turn is linked to fewer gestational weeks (i.e. prematurity) as compared to black race, which was linked to higher levels of hair cortisol, presence of preeclampsia and, in turn, to fewer gestational weeks. Childhood trauma was associated with adverse infant outcomes, namely small size for gestational age at birth. Moreover, this relationship was related to perceived social stress in adulthood, suggesting that those who experienced certain types or magnitude of childhood trauma may experience increased social stressors in adulthood that can also influence risk for adverse birth outcomes. Further examination of this connection is warranted.

Because the proposed approach is exploratory in nature, we emphasize that these results are tentative, with the purpose of contributing to the discussion surrounding adverse outcome pathways. Formal analyses based on the patterns discovered using PC-KCI need to be conducted with new data and approaches to confirmatory hypothesis testing, such as preplanned and pre-registered modelling approaches.

With proper cautions, this method and other graph-learning methods can be applied to a wide range of clinical and epidemiological datasets to gain insight on the relationships between suspected risk factors for clinical outcomes, and to improve the process of choosing pathways for further study. We propose that probabilistic graphical model algorithms could lead to more effective discovery of those physiological and psychosocial mechanisms responsible for complex clinical outcomes.

### Limitations

Statistical associations between two variables in prospective cohort studies are generally induced by direct causation (X -> Y), in which one variable causes the other, or confounding (X<- Z -> Y), in which there is a third, unmeasured variable that causes two measured variables. Statistical methods for distinguishing between these cases are limited and often controversial within the statistical community. For this reason, we proceed with caution in making causal claims based solely on observational data. However, the absence of statistical association generally indicates that two variables are not causally linked, given that there are enough data to detect an association.

The independence tests are probabilistic with an attendant degree of risk of false positives and false negatives. PC-KCI is likely to be too conservative, with a higher chance of false negatives or missing edges between variables in the graph. For analyses based on a priori theoretical models, a regression approach could produce somewhat different results, compared to the exploratory approach of PC-KCI. As with all statistical methods, external expertise is required to interpret the presence or absence of potential linkages.

Another issue is missingness in the data. Unfortunately, imputing missing values will only weaken dependence relationships, increasing the false negative rate on the edges, resulting in fewer edges than the shown model. Imputation methods combined with non-parametric graph learning are an important area for future research. In this analysis, we dropped observations only when a specific hypothesis test did not have sufficient information for a particular observation. That is, before performing each hypothesis test that PC required, we dropped variables for that specific test when they contained observations only for the requisite variables for missing values, but restored the dropped variables for subsequent independence tests.

In this work, we used a machine learning algorithm, PC-KCI, to model the statistical dependence relationships between multiple adverse pregnancy outcomes and risk factors. We analyzed the Measurement of Maternal Stress (MOMS) study data to provide an overview of the type of results that this approach can yield. By applying a probabilistic graphical model algorithm, PC-KCI, to data from a large, multi-center pregnancy cohort, we confirmed previous findings in the literature, identified measures that may have limited predictive value in the presence of other more proximal measurements, and suggested nuanced and novel pathways to preterm birth that may warrant further exploration.

## Supporting information

S1 Text KCI code. (PDF)

S2 Text Graph code. (PDF)
