# Supplementary material for: Using graph learning to understand adverse pregnancy outcomes and stress pathways
Source: PLoS One. 2019 Sep 30;14(9):e0223319. doi: 10.1371/journal.pone.0223319 (PMC6768465; doi:10.1371/journal.pone.0223319)
Supplement: S1 Text — (PDF) [file pone.0223319.s001.pdf]

```
#The program codes the KCI statistic
#Zhang 2012 Kernel-based conditional independence test...
setwd(".")
epsilon <- 0 #10^-2
eigThresh <- 10^-10
thres <- 1e-5

trProd <- function(A, B){
    stopifnot(ncol(A) == nrow(B))
    sum(sapply(1:nrow(A),
               function(ii){A[ii,] %*% B[,ii]}
               )
        )
}

trProdTrans <- function(A){
    sum(sapply(1:nrow(A),
               function(ii){A[ii,] %*% A[ii,]}
               )
        )
}

cholsky <- function(origMat){
    cholStatus <- try(u <- chol(origMat), silent = TRUE)
    cholError <- ifelse(class(cholStatus) == "try-error", TRUE, FALSE)
    if (cholError){
        u <- chol(origMat + 0.01 * diag(dim(origMat)[1]))
    }
    return(u)
}


gaussKernMat <- function(dat, theta){
    exp(as.matrix(-dist(dat, diag=TRUE, upper=TRUE)^2*theta/2))
}

ardKernel <- function(params, x, noise=TRUE){
    #This function is the ARD kernel
    #param = (variable precision params), signal var, model var)
    x <- as.matrix(x)
    xDim <- dim(x)
    prec_param <- exp(params[1:xDim[2]])
    sig_var <- exp(2*params[xDim[2] + 1])
    if(noise){
        mod_var <- exp(2 * params[xDim[2] + 2])
    } else { mod_var <- 0}
    kern <- sig_var *
        exp(-as.matrix(dist(
                 x %*% diag(1/prec_param, nrow=length(prec_param)),
                 diag=TRUE, upper = TRUE)^2/2
```

```
                 )
            ) + (mod_var + epsilon) * diag(xDim[1])
    return((kern+t(kern))/2)
}

ardGrad <- function(params, x, coord){
    xDim <- dim(x)
    prec_param <- exp(params[1:xDim[2]])
    sig_var <- exp(2*params[xDim[2] + 1])
    mod_var <- exp(2*(params[xDim[2] + 2]))
    if(coord <= xDim[2]){
    kern <-  #sig_var *
        exp(-as.matrix(dist(
                 x %*% diag(1/prec_param, nrow=length(prec_param)),
                 diag=TRUE, upper = TRUE)^2/2
                 )
            )
    out <- kern *
        as.matrix(dist(x[,coord],
                       diag=TRUE, upper=TRUE)/prec_param[coord])^2
    return(out)
    }
    if(coord == xDim[2]+1){
        kern <-  sig_var *
            exp(-as.matrix(dist(
                     x %*% diag(1/prec_param, nrow=length(prec_param)),
                     diag=TRUE, upper = TRUE)^2/2
                     )
                )
        return(2 * kern)
    }
    if(coord == xDim[2]+2){
        return(2 * mod_var * diag(xDim[1]))
    }
}


gplogLik <- function(params, x, y){
###changing to make more stable
    xDim <- dim(x)
    sig_var <- exp(2*params[xDim[2] + 1])  ###  Check this out
    x <- as.matrix(x)
    y <- as.matrix(y)
    xDim <- dim(x)
    yDim <- dim(y)
    kern <- ardKernel(params, x)
    L <- chol(kern)
    alpha <- solve(kern, y,
                   system=signature(a = "dppMatrix", b = "dsparseMatrix"))
    0.5 * sum(diag(t(y) %*% alpha)) +
```

```
        yDim[2]*(0.5 * log(sig_var) + sum(log(diag(L)))) +
        0.5 * xDim[1] * yDim[2] * log(2*pi)
}

gplogLikGrad <- function(params, x, y){
    paramNum <- length(params)
    yDim <- dim(y)
    out <- rep(NA, paramNum)
    kern <- ardKernel(params, x)
    alpha <- solve(kern, y,
                   system=signature(a = "dppMatrix", b = "dsparseMatrix"))
    W <- yDim[2] * solve(kern, system=signature(a = "dgCMatrix")) -
        tcrossprod(alpha)
    for(itr in 1:paramNum){
        out[itr] <- sum((W * ardGrad(params, x, itr)))/2
    }
    return(out)
}



kciTest <- function(x, y, z=NULL, theta=NULL){
    x <- as.matrix(x)
    y <- as.matrix(y)
    x <- scale(x) #scale standardizes columns
    y <- scale(y)
    nobs <- dim(x)[1]
    H <- diag(nobs) - matrix(1, nrow=nobs, ncol=nobs)/nobs

    if(is.null(theta)){
        if(nobs <= 200){
            width <- 1.2
        } else if(nobs > 1200){
            width <- 0.4
        } else {width <- 0.7}
        theta <- 1/width^2
    }


    if(is.null(z)){
        kernMatX <- gaussKernMat(x, theta)
        kernMatY <- gaussKernMat(y, theta)

        kx <- H %*% (kernMatX %*% H)
        ky <- H %*% (kernMatY %*% H)

        test.stat <- sum(diag(kx %*% ky))/nobs
        th.mean <- sum(diag(kx)) * sum(diag(ky)) / nobs^2
        th.var <- 2 * trProd(kx, kx) * trProd(ky, ky) / nobs^4
    } else{
```

```
z <- as.matrix(z)
z <- scale(z)
zDim <- dim(z)[2]

theta <- 1/(width^2 * zDim)

kernMatXd <- gaussKernMat(cbind(x,z/2), theta) #changed to test
kxd <- H %*% (kernMatXd %*% H)

kernMatY <- gaussKernMat(y, theta)
ky <- H %*% (kernMatY %*% H)

eigx <- eigen(kxd, symmetric = TRUE)
eigy <- eigen(ky, symmetric = TRUE)

eigx_keep <- eigx$value >= thres * eigx$value[1]
eigy_keep <- eigy$value >= thres * eigy$value[1]

mapx <- eigx$vectors[,eigx_keep] %*%
    diag(sqrt(eigx$values[eigx_keep]), nrow=sum(eigx_keep))
mapy <- eigy$vectors[,eigy_keep] %*%
    diag(sqrt(eigy$values[eigy_keep]), nrow=sum(eigy_keep))

init_eta <- log(width * sqrt(zDim))
init_params <- c(rep(init_eta, zDim), 0, log(sqrt(0.1)))
opt_x <- optim(init_params, gplogLik, gplogLikGrad,
               x=z, y=2*sqrt(nobs) * mapx/sqrt(eigx$value[1]))
opt_y <- optim(init_params, gplogLik, gplogLikGrad,
               x=z, y=2*sqrt(nobs) * mapy/sqrt(eigy$value[1]))
#opt_x <- Rcgmin(init_params, gplogLik, gplogLikGrad,
#               x=z, y=2*sqrt(nobs) * mapx/sqrt(eigx$value[1]))
#opt_y <- Rcgmin(init_params, gplogLik, gplogLikGrad,
#               x=z, y=2*sqrt(nobs) * mapy/sqrt(eigy$value[1]))
opt_hyp_x <- opt_x$par
opt_hyp_y <- opt_y$par

Px <- (diag(nobs) - ardKernel(opt_hyp_x, z, FALSE) %*%
       solve(ardKernel(opt_hyp_x, z)))
Py <- (diag(nobs) - ardKernel(opt_hyp_y, z, FALSE) %*%
       solve(ardKernel(opt_hyp_y, z)))

kxdz <- Px %*% (kxd %*% t(Px))

kyz <- Py %*% (ky %*% t(Py))

test.stat <- trProd(kxdz, kyz) #/ nobs

eigx <- eigen(kxdz, symmetric = TRUE)
eigy <- eigen(kyz, symmetric = TRUE)
```

```
        thrsX <- sum(eigx$values >= eigThresh)
        thrsY <- sum(eigy$values >= eigThresh)

        mapx <- eigx$vectors[,1:thrsX] %*%
            diag(sqrt(eigx$values[1:thrsX]), nrow=thrsX)
        mapy <- eigy$vectors[,1:thrsY] %*%
            diag(sqrt(eigy$values[1:thrsY]), nrow=thrsY)
        w <- do.call(cbind,  #stacking M_t
                     lapply(1:nobs,
                            function(t) as.vector(mapx[t,] %*% t(mapy[t,]))
                            )
                     )
        #trww <- trProdTrans(w)
        prod_w <- crossprod(w)
        sq_prod_w <- crossprod(prod_w)
        trww <- sum(diag(prod_w))
        th.mean <- trww #/ nobs
        sq_trww <- sum(diag(sq_prod_w))
        th.var <- 2 * sq_trww #/ nobs ^ 2
    }
    shape = th.mean^2 / th.var
    scale = th.var / th.mean
    p.val <- pgamma(test.stat, shape = shape, scale = scale,
                    lower.tail=FALSE) #gamma approx
    return(p.val)
}

kciWrap <- function(x,y,S, suffStat){
    compObs <- complete.cases(suffStat[,c(x,y,S)])
    compDat <- suffStat[compObs,]
    if (length(S) == 0) {
        error <- try(res <- kciTest(compDat[,x], compDat[,y]))
        if(class(error) == "try-error") res <- NA
    } else {
        error <- try(res <- kciTest(compDat[,x], compDat[,y], compDat[,S]))
        if(class(error) == "try-error") res <- NA
    }
    res
}

neighbors <- function(outcome, data, alpha, verbose=FALSE){
    nfeats <- dim(data)[2]
    rfeats <- 1:nfeats
    target <- nfeats + 1
    dat <- cbind(data, outcome)
    for(csize in 0:(nfeats-1)){
        if(length(rfeats) - 1 < csize){
            return(rfeats)
            break
        }
```

```
        current <- rfeats
        for(tvar in current){
            condvars <- rfeats[tvar != rfeats]
            if(length(condvars) != 1){
                condsets <- combn(condvars, csize)
            } else {
                condsets <- as.matrix(condvars)
            }
            ncondsets <- dim(condsets)[2]
            for(itr in 1:ncondsets){
                if(verbose) cat("y=", tvar, ", S=",condsets[,itr],", pvalue= ")
                pval <- kciMix(target, tvar, condsets[,itr], dat)
                ##pval <- myCItest(target, tvar, condsets[,itr], dat)
                if(verbose) cat(pval, "\n")
                if(pval >= alpha & !is.na(pval)){
                    rfeats <- rfeats[rfeats != tvar]
                    break
                }
            }
            if(verbose) cat("Remaining: ", rfeats, "\n")
        }
    }
}

myCItest <- function(x,y,S, suffStat){
    compObs <- complete.cases(suffStat[,c(x,y,S)])
    suffStat <- suffStat[compObs,]
    if (length(S) == 0) {
        res <- cor.test(suffStat[,x],suffStat[,y])$p.value
    } else {
        resx <- resid(lm(suffStat[,x] ~ as.matrix(suffStat[,S])))
        resy <- resid(lm(suffStat[,y] ~ as.matrix(suffStat[,S])))
        res <- cor.test(resx, resy)$p.value
    }
    res
}

kciMix <- function(x,y,S, suffStat){
    if (length(S) == 0) {
        res <- myCItest(x,y,S, suffStat)
        if(res > alpha) { ### p-value hard coded
            res <- kciWrap(x,y, S, suffStat)
            }
    }
    else if (myCItest(x,y,NULL, suffStat) <= alpha){ ### p-value is hard coded for
PCalg
        res <- myCItest(x,y, S, suffStat)
    }
    else {
        res <- kciWrap(x,y,S, suffStat)
```

```
        if(is.na(res)) {
            res <- myCItest(x,y,S, suffStat)
        }
    }
    return(res)
}
```
