# Supplementary material for: Using graph learning to understand adverse pregnancy outcomes and stress pathways
Source: PLoS One. 2019 Sep 30;14(9):e0223319. doi: 10.1371/journal.pone.0223319 (PMC6768465; doi:10.1371/journal.pone.0223319)
Supplement: S2 Text — (PDF) [file pone.0223319.s002.pdf]

```
require(pcalg)
source("kci.R")


dat <- read.csv(file="moms201711-1.csv", na.string=c("NA"))
labs <- names(dat)

alpha <- 0.05
moms.skel05 <- skeleton(dat, indepTest = kciMix, labels=labs, NAdelete = TRUE,
                  alpha=alpha, verbose=TRUE)
save(moms.skel05, file = "momsSkelOutputMix.RData")

alpha <- 0.01
moms.skel01 <- skeleton(dat, indepTest = kciMix, labels=labs, NAdelete = TRUE,
                  alpha=alpha, verbose=TRUE)
save(moms.skel05, moms.skel01, file = "momsSkelOutputMix.RData")

g05 <- moms.skel05@graph
g01 <- moms.skel01@graph

edgeList <- function(graph){
    el <- showEdgeList(graph)
    out <- data.frame(graph@nodes[el$undir[,1]], graph@nodes[el$undir[,2]])
    names(out) <- c("from", "to")
    out
}

edgeList01 <- data.frame(edgeList(g01), in01 = 1)
edgeList05 <- data.frame(edgeList(g05), in05 = 1)
elAll <- merge(edgeList01, edgeList05, by = c("from", "to"), all=TRUE)
names(elAll) <- c("var1", "var2", "in01", "in05")

#elAll$var1[which(elAll$var1 %in% c("White", "Black", "Other_race"))] <- "Race"
#elAll$var2[which(elAll$var2 %in% c("White", "Black", "Other_race"))] <- "Race"
#racerace <- which(elAll$var1 == "Race" & elAll$var2 == "Race")
#write.table(elAll[-racerace,], row.names = FALSE, file = "momsKciMixInt.txt", quote
= #FALSE)
```
